# De Novo Synthesis of VP16 Coordinates the Exit from HSV Latency In Vivo

**DOI:** 10.1371/journal.ppat.1000352

**Published:** 2009-03-27

**Authors:** Richard L. Thompson, Chris M. Preston, Nancy M. Sawtell

**Affiliations:** 1 Department of Molecular Genetics, Microbiology, and Biochemistry, University of Cincinnati School of Medicine, Cincinnati, Ohio, United States of America; 2 Medical Research Council Virology Unit, Glasgow, Scotland, United Kingdom; 3 Department of Pediatrics, Division of Infectious Diseases, Cincinnati Children's Hospital Medical Center, Cincinnati, Ohio, United States of America; Princeton University, United States of America

## Abstract

The mechanism controlling the exit from herpes simplex virus latency (HSV) is of central importance to recurrent disease and transmission of infection, yet interactions between host and viral functions that govern this process remain unclear. The cascade of HSV gene transcription is initiated by the multifunctional virion protein VP16, which is expressed late in the viral replication cycle. Currently, it is widely accepted that VP16 transactivating function is not involved in the exit from latency. Utilizing the mouse ocular model of HSV pathogenesis together with genetically engineered viral mutants and assays to quantify latency and the exit from latency at the single neuron level, we show that in vivo (i) the VP16 promoter confers distinct regulation critical for viral replication in the trigeminal ganglion (TG) during the acute phase of infection and (ii) the transactivation function of VP16 (VP16TF) is uniquely required for the exit from latency. TG neurons latently infected with the VP16TF mutant in1814 do not express detectable viral proteins following stress, whereas viruses with mutations in the other major viral transcription regulators ICP0 and ICP4 do exit the latent state. Analysis of a VP16 promoter/reporter mutant in the background of in1814 demonstrates that the VP16 promoter is activated in latently infected neurons following stress in the absence of other viral proteins. These findings support the novel hypothesis that de novo expression of VP16 regulates entry into the lytic program in neurons at all phases of the viral life cycle. HSV reactivation from latency conforms to a model in which stochastic derepression of the VP16 promoter and expression of VP16 initiates entry into the lytic cycle.

## Author Summary

Herpes simplex virus (HSV) establishes life-long latent infections in sensory neurons of the human host. Periodically, HSV exits latency in an infected neuron and is transported to the body surface where it replicates, leading to recurrent disease and infection of new hosts. We do not currently understand how entry into the lytic cycle is blocked in neurons and latency is established. Nor do we know how, at some time in the future, the lytic program becomes activated in the one or two latently infected neurons which characterize a reactivation event. In tissue culture cells, and by analogy in cells at the body surface, the HSV replication program is initiated by the interaction of a virion protein, VP16 (brought in with the virus as a protein), with host cell factors. Here we show that the de novo synthesis of VP16 is required for efficient viral replication during the acute phase of infection in neurons. This indicates that latency is favored because VP16 may not be transported efficiently to the nerve cell nucleus. Once latency is established, the de novo expression of VP16 is an absolute and very early requirement for the exit from the latent state. Our data support a model of HSV reactivation in which the stochastic derepression of the VP16 promoter and resulting expression of VP16 starts the viral lytic program.

## Introduction

Primary infection with herpes simplex virus (HSV), universally the result of close contact with an infected individual, is accompanied by dissemination of viral genomes into the host nervous system. Although symptoms of the primary infection usually resolve, large numbers of viral genomes remain in a transcriptionally repressed state within neurons of sensory ganglia and the brain for the life of the infected individual [1]. Periodically, stimulated by various stressors, latency is exited and infectious virions are generated in a small number (<0.05%) of latently infected neurons [2]–[5] which transport virus back to the body surface through innervating axons. Although individual neurons supporting lytic viral replication do not survive this process [4]–[6], the large reservoir of latently infected neurons allows this cycle to occur repeatedly which is the mechanism of transmission and the cause of serious sequellae including blindness and encephalitis. That 70–90% of the human population worldwide is now infected is a testament to the efficacy of this strategy [7]. There is currently no way to either eliminate latent virus or to prevent the exit from latency and no effective vaccine to protect the uninfected, thus transmission rates remain high. To date, the molecular mechanisms regulating reactivation from latency remain unclear. Identifying the interactions between the neuron and latent viral genome that result in the exit from latency is critical toward progress in understanding and ultimately controlling this complex process.

In a striking case of parallel evolution, most DNA viruses employ strong enhancers to promote the transcription of the earliest viral genes [8]. HSV differs from other DNA viruses including most other herpesviruses in that transcription of its immediate early (IE) genes is principally dependent on a protein component of the virion that is a potent transcriptional activator [9],[10]. This multifunctional late gene protein, VP16 (VMW65, α-TIF, UL48), interacts with host cell proteins including HCF-1, a cell cycle regulator, and Oct-1, a POU domain transcription factor, to form the VP16 induced complex (VIC) that binds to TAATGARAT elements present in the five HSV-1 immediate early gene promoters [11]–[14]. Considering the complex in vivo life cycle of HSV, the dependence on a structural protein produced late in the infectious cycle to initiate transcription from the viral genome presents a conundrum. How can the latent viral genome initiate the transcription of lytic phase genes in the absence of this crucial transcriptional activator? Studies in the early 1990's led to the dogma that VP16 is simply not involved in reactivation [15]–[17] and that its function in initiating the lytic cycle is fulfilled by another viral function or a host cell factor.

There have been two long-standing hypotheses regarding the initiation of the lytic cycle during reactivation from latency. The first hypothesis proposes that the viral IE gene ICP0 initiates reactivation from latency [18]–[21]. The second proposes that viral early gene expression and DNA replication precedes and is required for efficient IE gene expression during reactivation from latency [22],[23]. In these studies, reactivation was evaluated using axotomized and explanted ganglia. Although this assay has been widely utilized, it has become increasingly clear that explant reactivation does not model HSV reactivation as it occurs in vivo [24],[25]. In hindsight, this is not a surprising finding, in that axotomized and explanted neurons rapidly undergo radical transcriptional changes, including apoptosis [24],[26],[27]. Recent reexamination of these hypotheses using in vivo reactivation and single neuron level approaches have demonstrated that the exit from latency does not require either a viral DNA pre-amplification step [28] or functional ICP0 [25]. An important clue as to how exit from latency is regulated came from the analysis of a viral mutant termed ΔTfi in which a 350 bp region of the ICP0 promoter, which includes the TAATAGARAT element through which VP16 transactivates this IE gene, is deleted. Although this mutant reactivates with wild type kinetics in explant assays [25],[29], in vivo it is severely impaired in its ability to reactivate, suggesting that transactivation by VP16 may indeed be critical in the regulation of reactivation in vivo [25].

Here we report results from experiments designed to test the hypothesis that VP16 regulates the exit from latency. Our studies support the hypothesis that in elegant simplicity, the major coordinator of IE gene expression and tegument protein, VP16, functions to regulate entry into the lytic program at all phases of the viral life cycle. We find that in vivo (i) the VP16 promoter confers distinct regulation critical for viral replication in the trigeminal ganglion, and (ii) VP16 transactivating function is required for reactivation from latency. Importantly, that VP16 transactivation function (VP16TF) is required very early in the exit from latency is supported by (i) failure of latent viral genomes to enter the lytic cycle (as defined by expression of lytic viral protein) uniquely in the absence of VP16TF (ICP0 null, viral thymidine kinase null, and tsICP4 mutants do exit latency), and (ii) the restoration of reactivation competency of ΔTfi by replacement of the TAATGARAT element. In the nervous system, de novo expression of VP16 from the latent viral genome allows VP16 to coordinate the expression of the viral IE genes and thereby initiate the productive lytic cycle.

## Results

### The VP16 promoter has unique regulatory properties in neurons in vivo

HSV initiates the viral lytic cycle under two distinct conditions, (i) following infection of a cell by the virion, and (ii) from the latent viral genome. In the first case, the lytic cycle is engaged through coordinated activation of the viral IE genes by the virion associated transactivator, VP16 [11]–[14]. How the lytic cycle is initiated from the latent genome remains unknown, although it is reasoned that VP16, expressed with late kinetics during the lytic cycle, does *not* supply this function [15], [16], [18], [30]–[33].

**Figure 1:**
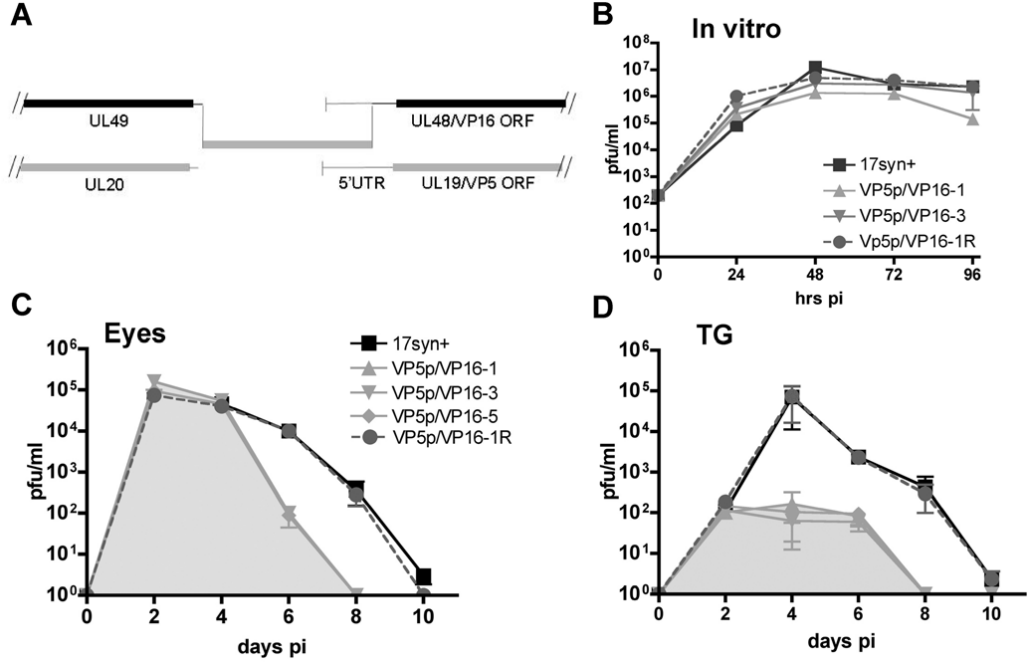
Replication of VP5p/VP16 mutants in vitro and in vivo. (A) A diagram of the construction of the VP5p/VP16 mutants is shown. The VP16 promoter and 5′UTR sequences were replaced with those of another gene expressed with leaky late kinetics, the VP5 gene, as detailed in Methods. (B) RSC were infected with mutants VP5/VP16-1 and -3, the genomically restored isolate (VP5p/VP16-1R), and wild type HSV-1 strain 17Syn+ at an moi of 0.0004 pfu/cell. At the indicated times, 3 plates infected with each virus were harvested and assayed independently for virus content as detailed in Methods. (C,D) Mice were infected as detailed in Methods and, at the indicated times pi, tissues from three mice from each group were assayed for virus content. The grey shading in C and D indicates the regions employed to calculate the areas under the curves for the VP5p/VP16 mutants.

ICP0 null mutants can exit latency (demonstrated by the detection of lytic viral protein expression), however, progression to lytic virus production (reactivation) does not occur [25]. In addition, a mutant in which the VP16 binding site has been deleted from the ICP0 promoter also fails to reactivate in vivo. Together, these findings raise the possibility that VP16 may play an unexpected role in the regulation of IE genes very early in the exit from latency. If this were the case, the regulation of VP16 must be distinct in this context, with the protein expressed as a very early event and not as a standard leaky late gene. To test this, we asked whether another viral promoter of equivalent strength and kinetic class [34]–[36] could confer “proper” regulation of VP16 in vivo. The VP5 promoter was selected since replacement of this viral promoter with that of VP16 has been reported previously and no measurable effect on the ability of the virus to replicate in vivo, either at the surface or in the nervous system was observed [37]. Thus the converse mutant in which the VP16 promoter/5′utr was replaced with that of VP5 was generated as detailed in methods. A diagram of this mutant is shown in Figure 1A. Three independently derived viral mutants were characterized in vitro and in vivo. Levels of VP16 mRNA in rabbit skin cells (RSC) infected with mutant VP5p/VP16 were not reduced compared to 17syn+ as quantified by northern blot analysis at 6, 8 and 12 hr pi (not shown). Standard single (not shown) and multi-step replication kinetic analysis in RSC revealed no alterations when compared to the parental strain 17syn+ or the genomically restored mutant VP5p/VP16-1R (Figure 1B). In order to determine the effect, if any, of this promoter exchange on viral replication in vivo, five groups of 16 mice each were inoculated on scarified corneas with 1×10^5^ pfu of either VP5p/VP16-1,-3, -5, VP5p/VP16-1R, or 17syn+. Titers of infectious virus in the eyes and trigeminal ganglia (TG) were determined independently in three mice from each group on days 2, 4, 6, 8, and 10 pi. The total amount of infectious virus detected in the eyes during the acute stage of infection was not different among the viruses compared, (area under the curve (AUC) = 292, 261 vs. 250,013 or 281, 982, respectively) (Figure 1C). Note also that the peak viral replication occurring in the eyes on day 4 pi was not different (p = 0.65; ANOVA ). In contrast, total infectious virus detected during the acute stage in the TG was more than 200 fold reduced for the VP5p/VP16 mutants compared to the parental strain or the genomically restored isolate (AUC = 628 vs. 155,237 or 148, 810) and the peak viral titers detected on day 4 in VP5/VP16 infected TG was more than 2 orders of magnitude lower than those detected in 17syn+ or VP5/VP16-1R infected TG (Figure 1D). The viral feedback loop between the surface and the ganglion is well documented [38],[39]. The decline in viral titers in the eyes of VP5p/VP16 infected mice (days 6–8) most likely resulted from the absence of significant replication in the ganglia and transport of virus back to the eye as described previously [39]. Importantly, the high viral titers generated by mutant VP5p/VP16 in RSC and on the corneal surface in vivo confirm the infectious nature of the virions, which would not be the case if levels of VP16 in the tegument were deficient [30],[40],[41]. The VP16 protein produced during infection with this mutant is fully functional and would be anticipated, if it were indeed efficiently transported to the neuronal cell body, to initiate lytic viral infection in the neuron. However, the replication of the VP5/VP16 mutants in TG is severely impaired, although viral DNA is transported to the ganglion as determined by real-time PCR assay (not shown). This strongly suggests that viral replication in neurons is in fact *not* initiated by VP16 protein transported from the surface, but rather by its synthesis in the infected neuron de novo. The profound selective loss of replicative capacity in the TG of mice infected with the VP5p/VP16 mutant provides the first evidence that the VP16 promoter is unique in its ability to regulate gene expression in the nervous system and supports the hypothesis that VP16, through distinct regulation in the TG neurons, could play an important role in exiting latency.

### Altered expression kinetics of the VP16 promoter in neurons in vivo

The possibility that de novo expression of VP16 may be required in neurons during both the acute stage of infection and during reactivation is suggested when considering collectively (i) the well documented requirement for VP16 transactivating function during the acute infection in TG [16],[30] (presumably for entry into the lytic cycle) , (ii) the inadequacy of leaky late expression of VP16 from the VP5 promoter to support lytic viral replication (reported here) and (iii) evidence from another α-herpes virus that viral nucleocapsids arrive at the neuronal cell body largely devoid of VP16 [42],[43]. Framed within conventional understanding of HSV gene regulation, the question to be asked is straightforward, namely is VP16 expressed as a late gene, as demonstrated in tissue culture or is VP16 expressed with distinct kinetics in neurons? The concept of cascade gene regulation [44] and the kinetic class of viral promoters during viral lytic cycle are fundamental to how we view this process. However, these criteria were developed from en masse analyses of synchronously infected cells of uniform type in the presence of drug blockades. This experimental format cannot be recapitulated in vivo. One approach to evaluating promoter activity in vivo is through the generation of viral promoter/reporter mutants [45]–[55]. For this purpose, a VP16 promoter/beta-galactosidase gene (LacZ) reporter mutant was generated as detailed in methods and utilized to ask whether activation of the VP16 promoter in neurons is consistent with conventional leaky late gene expression. If this were the case, then VP16 promoter activity would be anticipated only in neurons expressing lytic viral protein.

**Figure 2:**
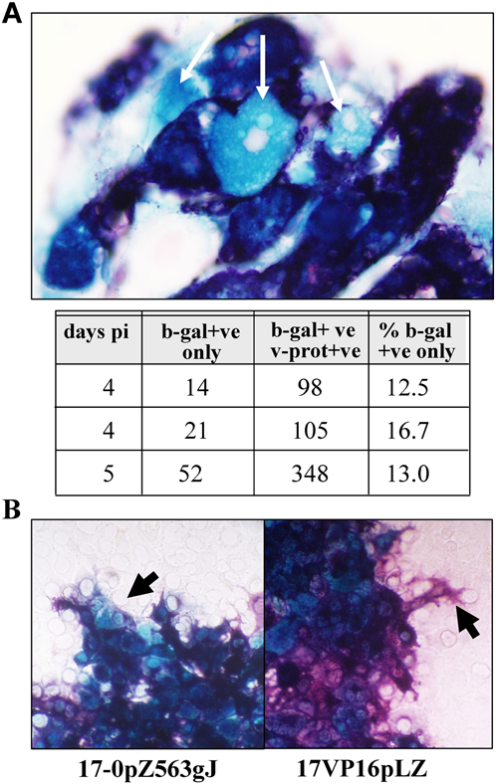
Activation of the VP16 promoter in sensory neurons during acute infection. (A) Mice were infected with 17VP16pLZ. At the indicated days pi, TG were removed and sequentially processed for the detection of b-gal activity (blue) and viral proteins (purple), as detailed in Methods. A photomicrograph of a focus of infection in a TG is shown. White arrows indicate neurons with b-gal activity with little or no detectable viral protein. Counts of neurons positive for only b-gal or b-gal plus viral protein are indicated below the micrograph. (B) Photomicrographs of viral plaques on RSC monolayers infected at low moi with 17-0pZ563gJ and 17VP16pLZ are shown. The monolayers were stained to detect b-gal and viral proteins. At regions at the edge of the plaques formed by 17-0pZ563gJ cells expressing b-gal (blue) with little or no evidence of viral protein expression were evident (arrow). Regions at the edge of plaques formed by 17VP16pLZ show cells staining positive for viral proteins (purple) with little or no staining for b-gal evident (arrow).

TG from mice inoculated with 2×10^5^ PFU of 17VP16pLZ were harvested on days 4 and 5 pi and processed sequentially for in situ E. coli beta-galactosidase (b-gal) activity and for HSV proteins as detailed previously [25],[28],[55]. Figure 2A shows two populations of neurons evidencing activity from the viral genome. In the majority of these neurons (464/551, 86%), VP16 promoter activity was co-localized with lytic viral proteins. However, in 13–16% of positive (infected) neurons, the VP16 promoter was active in the absence of detectable lytic viral proteins. Even if very low and undetectable levels of viral proteins are present in these neurons, the findings indicate that in neurons, activation of the VP16 promoter can precede expression of significant levels of viral proteins, an expression pattern inconsistent with our understanding of late gene expression.

When examined in infected RSC, the pattern of expression of the VP16 promoter was consistent with late kinetics in that at either high or low multiplicity of infection (moi), b-gal activity was detected only in cells in which viral proteins were also detected. The asynchrony of low moi infection more closely represents infection in vivo and in this case plaques formed by 17VP16pLZ were ringed by cells expressing viral proteins but little or no b-gal activity (Figure 2B). We examined the expression of an IE gene promoter/reporter virus, 17-0pZ56gJ [55] using this same assay and observed that plaques were now ringed by cells expressing b-gal with very low levels of viral proteins present, as would be expected for a promoter activated at the initiation of the lytic cycle. These findings support the hypothesis that the regulation of VP16 in vivo is dependent on cell type and different from that seen in vitro.

### VP16 is expressed during reactivation in the absence of ICP0, ICP4, and viral DNA synthesis

We have reported previously that two viral functions (ICP0 and viral DNA synthesis) considered to play critical roles in the initiation of reactivation from latency, are in fact not required for lytic viral protein expression following a reactivation stimulus in vivo [25],[28]. These functions are, however, required for progression to infectious virus production. This knowledge provides the opportunity to ask whether VP16 is expressed in the absence of ICP0 function and in the absence of viral DNA replication following a reactivation stimulus. If VP16 is not present, it would suggest that this protein is not likely to be initiating entry into the lytic cycle. If, however, VP16 is detected, it would be consistent with an early role and reveal that in the context of reactivation, the expression of VP16 is not dependent upon either ICP0 function and/or viral DNA replication, both of which play a role in the regulation of late gene expression in cultured cells.

The exit from latency in vivo is highly a controlled process, restricted to a very small percentage of those neurons latently infected per event. Despite this, the number of neurons exiting latency and the number of neurons expressing VP16 can be quantified using whole ganglion immunohistochemistry (WGIHC), an assay that has been validated to provide a precise quantitative readout on the number of neurons expressing lytic viral proteins within a ganglion [56] Groups of mice were inoculated with either dl1403 (an ICP0 null mutant [57]) or 17tBTK- (a thymidine kinase negative mutant [24]). In the absence of the viral thymidine kinase (TK) function, viral DNA synthesis and replication in neurons are severely impaired. This gene is required for reactivation [58]–[61] but not for entry into the lytic cycle from the latent viral genome [28]. The deficit in each of these mutants results in significantly reduced total latent viral DNA [62]–[64] and numbers of latent infections in the TG, [25],[28],[65],[66], which in turn, reduces the number of neurons which exit latency [25],[28],[55],[56],[66]. Nevertheless, VP16 protein was detected at 22 hrs post hyperthermic stress (HS) in neurons in ganglia from mice latently infected with both of these mutants (3/27 and 9/20 in 17tBTK- and dl1403 infected ganglia, respectively). Analysis of the second TG of each pair with the anti-HSV antibody revealed no difference compared to the number of neurons in which VP16 was detected (4/27 and 8/20 in 17tBTK- and dl1403 infected ganglia), and numbers similar to our previous reports [25],[28]. Viral protein expressing neurons were not detected in uninduced latently infected ganglia (0/18). The number of neurons expressing VP16 within a positive ganglion ranged from 1–3, and these numbers were not different than those detected when using the anti-HSV antibody, which detects lytic viral proteins from IE, early (E) and late (L) kinetic classes. Likewise, following HS of mice latently infected with tsK+, a 17syn+ based mutant with a temperature sensitive mutation in the essential viral IE transactivator ICP4 [67], VP16 was expressed in rare neurons. The number of neurons in which latency was established with this mutant was very low (2.7%), yet VP16 was detected in rare neurons post stress (2 neurons in 2/30 ganglia were positive). This number is consistent with the frequency of reactivation observed in ganglia infected with wild type 17syn+ in which there were similar low levels of latency (1/31 positive) [56]. These findings indicate that independently, neither ICP0, ICP4, nor viral DNA replication is required for VP16 expression during reactivation in vivo.

In order to examine a larger population of neurons exiting latency, a chemical blockade of viral DNA replication was used to investigate the influence of viral DNA replication on the expression of VP16 in 17syn+ infected neurons following a reactivation stimulus. As shown previously, acyclovir (ACV) blocked detectable infectious virus production during reactivation in vivo [28],[68]. Infectious virus was not detected at 22 h post HS in the 14 TG tested (0/14). Ganglion pairs from an additional 15 mice from this group were harvested and examined using WGIHC. The number of neurons expressing lytic viral proteins of diverse kinetic classes was quantified in one ganglion from each pair and VP16 expression was quantified in the second ganglion from each pair. The number of neurons exiting latency, whether detected by the anti-HSV antibody (31 neurons/15 ganglion) or the antibody specific for VP16 (33 neurons/15 ganglion) was not different and similar to the numbers of neurons exiting latency previously reported [4],[24],[25],[55],[56],[66],[69]. As observed in 17tBTK- infected TG, blockade of viral DNA replication did not alter the expression of VP16.

### Mutants lacking the transactivation function of VP16: in1814 and 17VP16Δ422

**Figure 3:**
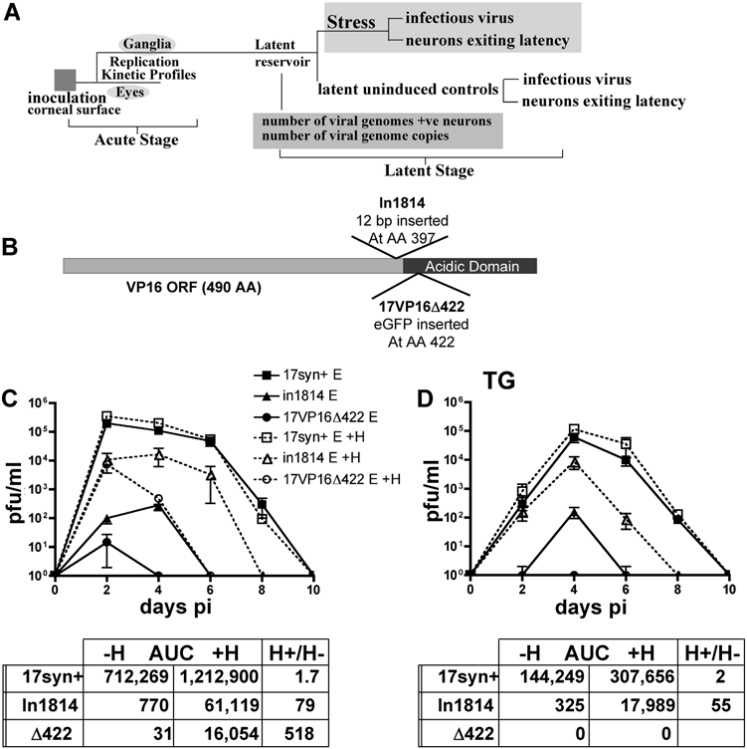
Replication kinetics of the VP16 transactivation mutants in1814 and 17VP16Δ422. (A) A schematic of the experimental design employed to biologically characterize the VP16 mutants is shown. (B) A diagram of the genomic structures of in1814 and 17VP16Δ422 is shown. (C,D) Mice were infected as detailed in Methods and, at the indicated times pi, tissues from three mice from each group were assayed for infectious virus. Solid lines represent titers obtained by standard plaque assay, whereas dashed lines represent the same samples titrated in the presence of 3 mM HMBA, as described in Methods. The area under the curve (AUC) was calculated for each curve, and the fold increase in AUC in the presence of HMBA is indicated below the graphs. Genomically restored isolates of both in1814 (1814R) and 17VP16Δ422 (17VP16Δ422R) were analyzed and found to be not different from the parental strain, 17syn+ (not shown).

VP16 is an essential multifunctional protein. However, mutations which impair the transactivation function of VP16 have been generated and characterized in vitro and in vivo. Mutant in1814 contains a 12 bp insertion that disrupts a domain required for the VP16 induced complex formation and thus the transactivation function of the protein [40],[70]. The carboxy-terminal acidic activation domain has been deleted in two mutants, V422 [41] and RP5 [30], both built in HSV-1 strain KOS. While these three mutants are phenotypically similar in vitro, important differences have been reported in their in vivo phenotypes. Despite the impaired replication reported for both in1814 and RP5 in mouse eyes and TG, in1814 established latent infections efficiently and reactivated in explant assays [16],[71],[72]. RP5 failed to accomplish either of these outcomes [30]. The in vivo phenotypic differences between HSV strains 17syn+ and KOS is a confounding issue [69],[73]. Therefore, mutant 17VP16Δ422 was constructed as detailed in methods. We utilized mutants in1814 and 17VP16Δ422 to evaluate the role of VP16 transactivation on reactivation in vivo as outlined in Figure 3A.

### In vivo replication kinetics

Groups of male Swiss Webster mice were inoculated as described in methods. Viral replication was evaluated on days 2,4,6,8 and 10 pi in tissues harvested from 3 mice from each inoculation group. On day 4 pi, 3–4 logs fewer pfu were detected in the eyes and TG of in1814 and 17VP16Δ422 infected mice compared to those infected with in8141R, 17VP16Δ422R, and 17syn+ (Figure 3C and 3D, and not shown). These results are in general agreement with previous reports [16],[30].

Interpretation of this result is complicated by the fact that at low moi (such as a plaque assay), mutants lacking the transactivation function of VP16 enter the lytic cycle inefficiently, leading to an underestimate the amount of virus present [40],[41],[74]. Several strategies have been utilized to overcome this problem, including VP16 expressing cell lines, and superinfection with a replication impaired virus [16],[30]. The addition of the cell differentiating agent, hexamethylene bisacetamide (HMBA), to cell cultures increases the plaquing efficiency of in1814 [74]. As shown in Figure 3C and 3D, the addition of HMBA to the culture medium revealed the presence of 100 and 500 fold more virus in in1814 and 17VP16Δ422 eye homogenates (day 2 pi), respectively. As anticipated, this compound had little effect on the plaquing efficiency of the parental strain, 17syn+ (1.8 fold increase in virus detected in homogenates from 17syn+ infected eyes). Differences between the two VP16 mutants, in1814 and 17VP16Δ422, to replicate in the TG were dramatic. Plaque assays performed in the presence of HMBA revealed that in1814 did replicate within the TG, although maximum titers are 17 fold lower than those achieved by wild type virus or 1814R (Figure 3D and not shown). In contrast, even in the presence of HMBA, infectious virus was not detected in 17VP16Δ422 infected TG, although ∼200 pfu were detected in the TG on day 4 pi when input titers of this mutant were increased (not shown). The importance of viral replication within the TG for achieving maximum numbers of latently infected neurons has been demonstrated [66]. That in1814 actually does replicate within TG is consistent with its ability to efficiently establish latent infections as well as reports that this mutant may retain some residual transactivating function [75].

### Quantification of the number of latently infected neurons and latent viral genome copy number profile in in1814-infected ganglia

**Figure 4:**
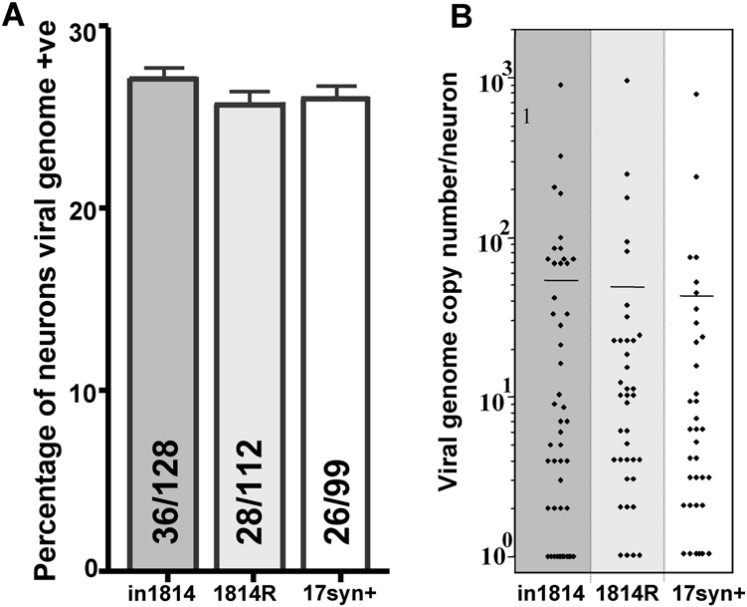
Mutant in1814 establishes latency as efficiently as genomically wild type isolates. Groups of mice were infected with strain 17Syn+, in1814, and the genomically rescued variant 1814R, as described in Methods. At 40 days pi, the ganglia of 3 mice per group were processed for single neuron PCR. Individual neurons were examined for the presence of the viral genome and the number of viral genomes present in positive neurons was determined using a quantitative PCR assay as detailed in Methods. (A) Shown is the percentage of neurons positive for the viral genome. The number of neurons positive for the viral genome over the number tested is shown in the histograms. (B) Each point on the scattergram represents the number of viral genomes present in an individual neuron. The horizontal bars are drawn at the mean value of genome copies per positive neuron.

In preliminary studies, 17VP16Δ422 was determined to establish latent infections, but at very low levels compared to 17syn+ (not shown). Thus it becomes impractical to study in vivo reactivation with this mutant because the efficiency of reactivation in vivo following HS is directly correlated with the number of latently infected neurons in the ganglion (r^2^ = 0.99) [56],[76]. Likewise quantification of the number of latently infected neurons in in1814 infected TG compared to the parental strain (17syn+) and rescue (1814R) is critical for interpreting the outcome of experiments to quantify viral reactivation. The number of latently infected neurons and the number of viral genomes within individual infected neurons in TG from 3 mice from each group was quantified using a single neuron PCR assay termed CXA [25],[28] [66] [77]. In this assay, ganglia stabilized by fixation are enzymatically dissociated and individual neurons from enriched neuronal fractions are harvested and analyzed by QPCR, providing information on both the frequency of latently infected neurons and the number of viral genome copies in the individual neurons analyzed. As anticipated from the results of preliminary experiments, similar numbers of latently infected neurons were observed in in1814, 1814R, and 17syn+ infected ganglia, 28%, 25% and 26%, respectively (Figure 4A). The number of viral genomes detected within individual latently infected neurons is shown in the scattergram (Figure 4B). No significant difference among the viral genome copy number profiles was observed (mean copy number = 56.5, 51.8 and 44.8, respectively p = 0.94; ANOVA).

### In1814 does not reactivate in vivo following HS

**Figure 5:**
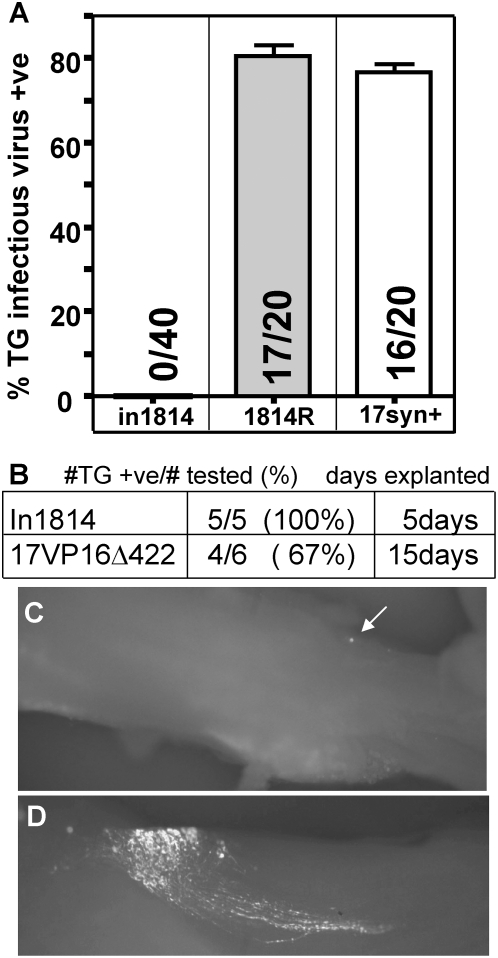
The mutant in1814 does not reactivate from latency in vivo but does reactivate in explanted ganglia. (A) Mice were infected as described in Methods. At 40 days pi, latently infected mice were induced to reactivate using HS, and TG were removed and assayed for infectious virus as detailed in Methods. The histograms represent the percentage of mice in which reactivated HSV was detected. The number of mice positive/tested is shown on the histograms. (B) Explant reactivation of ganglia latently infected with in1814 or 17VP16Δ422. TG from latently infected mice were axotomized and explanted. In1814 and 17VP16Δ422 infected TG were assayed for infectious virus on days 5 and 15 post explant, respectively. The GFP expression cassette in 17VP16Δ422 was used to detect gene expression from the viral genome during explantation. Exit from latency was observed as single GFP positive neuron at day 4 (C) which resulted in the spread of virus through the TG by day 6 (D).

At 40 days pi, mice from each group of infected mice were induced to reactivate in vivo using HS [5],[24]. At 22 hrs post treatment, mice were euthanized, ganglia were removed and homogenized and the homogenates assayed for infectious virus in the presence of 3 mM HMBA. In striking contrast to previous reports in which in1814 reactivated in explant reactivation assays, infectious virus could not be detected in any ganglia (0/20) from in 1814 infected mice induced to reactivate in vivo. However, infectious virus was detected at 22 hr post treatment in 17/20 (85%; p = 0.0002) and 16/20 (80%; p = 0.0002) of the TG pairs from mice infected with 1814R and 17Syn+ (Figure 5A).

HS has been utilized extensively and shown to reproducibly induce viral reactivation which peaks at ∼22 hrs post treatment using several laboratory strains [69] as well as 10 low passage clinical isolates (unpublished). However, it was possible that reactivation of in1814 was delayed compared to wild type virus. Infectious virus could not be detected in ganglia of in1814 infected mice 48 hrs post HS (0/20). These data demonstrate that in1814 did not reactivate to detectable levels in vivo in response to HS.

### In1814 and 17VP16Δ422 reactivate in vitro following explant of TG into culture

In order to confirm that ganglia from this group of in1814 infected mice would produce infectious virus when axotomized and explanted as previously reported [16],[17], the 10 TG from 5 mice latently infected with in1814 were either directly homogenized and assayed for infectious virus in the presence of HMBA or explanted and cultured for 5 days and then tested for infectious virus in the presence of HMBA (see methods). No virus was detected in TG homogenized directly upon removal but infectious virus was detected in 100% (5/5) of explanted TG, a finding similar to previous reports in which neurons were axotomized [16],[17] (Figure 5B). Similarly, the ability of ganglia from 17VP16Δ422 infected mice to reactivate in explant was tested. The presence of eGFP in this virus was used to monitor exit from latency and spread within the explanted ganglia over time. No GFP expression was detected in ganglia (0/6) at the time of explant, however within 4 days, a single GFP positive neuron was detected in 1/8 ganglia and by day 6 post explant, virus had spread within this TG (Figure 5C and 5D). After 15 days in explant, 17VP16Δ422 exited latency in 4/6 ganglia (Figure 5B). Ganglia were homogenized and plated in the presence of HMBA and infectious virus was recovered which was GFP positive and confirmed by southern blot to have the expected genomic structure (not shown).

### Latent viral genomes lacking VP16 transactivating function do not exit latency in vivo

**Figure 6:**
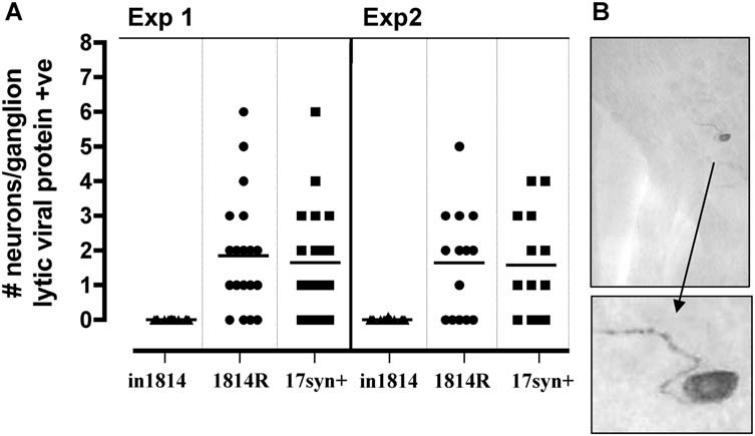
In1814 does not exit latency in vivo. (A) Mice latently infected with the indicated strains were subjected to HS, and whole TG were assayed for viral protein positive cells 22 hrs post stress. Each point in the scattergram represents the number of viral protein positive neurons detected in an individual TG. The horizontal bars are drawn at the mean. (B) A photomicrograph of a whole mounted ganglion processed for the detection of HSV-1 proteins 22 hr post HS shows a single neuron in which the latent genome has entered the lytic cycle. The brown precipitate evident in the cell body and axonal tract indicates the presence of viral proteins.

Reactivation from latency is functionally defined by the detection of infectious virus. To expand our understanding of the process of reactivation, we have developed a strategy for quantifying at the single neuron level the number of neurons which exit latency as evidenced by detectable lytic viral protein expression [4],[78]. This method is the first to allow us to partition the process of reactivation into stages, and to begin the assignment of viral and host cell functions critical for either entry into the lytic cycle or for progression to infectious virus production [25],[28]. In addition, this approach obviates the inherent problem of detection of reactivated virus when a mutant with low plaquing efficiency (such as in1814) is employed. At 40 days pi, additional mice from the groups detailed above were induced to reactivate in vivo using HS. Latently infected control mice and treated mice (at 22 hrs post treatment) were euthanized, the ganglia were removed and processed for the detection of lytic viral proteins using WGIHC as detailed previously [4],[78]. This method can reliably detect a single neuron exiting latency among the 10's of thousands in the ganglion. In the uninduced groups of animals, lytic viral protein expressing neurons were not observed (0/10, 0/8, and 0/9, in1814, 1814R and 17syn+ infected ganglia, respectively). This was not unexpected as we have previously shown that the level of “spontaneous” reactivation of strain 17syn+ in the latently infected Swiss Webster mouse TG is very low [4],[78]. Consistent with the detection of infectious virus above, ganglia from mice latently infected with either 1814R or 17syn+ contained neurons expressing lytic viral proteins at 22 hrs post induction, a total of 60 and 55 neurons, respectively in the ganglia examined (Figure 6A and 6B). In contrast, no lytic viral protein expressing neurons were detected in in1814 ganglia post induction (0/40). These findings indicate that in vivo, the VP16 transactivating function is required for HSV to exit the latent state and produce detectable viral proteins.

### The TAATGARAT element restores the wild type reactivation phenotype to an ICP0 promoter deletion mutant

If VP16 functions at the earliest stages of the initiation of reactivation, its role is likely to be the coordinated activation of immediate early genes through the TAATGARAT promoter elements. We reported previously that mutant ΔTfi, which contains a 350 base pair deletion in the ICP0 promoter including the TAATGARAT element, reactivates with wild type frequency and kinetics from ganglia axotomized and explanted [25],[29], but exhibits severely impaired reactivation in vivo [25]. To test the importance of the TAATGARAT to this phenotype, a mutant, ΔTfi+TAATGARAT, in which the TAATGARAT regulatory element was added back in its proper context to the ICP0 promoter of ΔTfi, was generated as detailed in methods and tested for its ability to reactivate in vivo.

Mice were inoculated with 4×10^6^ pfu of ΔTfiR or ΔTfi+TAATGARAT and at 40 days pi groups of latently infected mice were utilized to quantify the number of latent infections and to determine the in vivo reactivation frequency. There was no difference in either the number of neurons latently infected or the viral genome copy number profile in TG latently infected with ΔTfiR or ΔTfi+TAATGARAT (not shown). Infectious virus was not detected in latently infected uninduced TG from either ΔTfiR or ΔTfi+TAATGARAT infected mice, 0/5 and 0/5, respectively. However at 22 h post HS, infectious virus was detected in 41% (7/17) of TG from ΔTfiR and 44% (8/18) of TG from ΔTfi+TAATGARAT infected mice. Taken together, this finding and the clear requirement for VP16 transactivating function for the exit from latency in vivo, support the hypothesis that as during acute lytic infection, VP16 operates through the TAATGARAT element to regulate immediate early gene expression during reactivation.

### VP16 promoter is activated in neurons latently infected with in1814 following HS

The preceding experiments suggest an amplification feedback loop between VP16 and the IE gene products is required to exit latency. If this is true, expression of VP16 must occur very early, requiring that the VP16 promoter be activated de novo in contrast to the standard cascade of viral functions facilitating the activation of the viral leaky late promoters. With wild type HSV, once reactivation is initiated, the production of the viral IE transactivator proteins induces transcription from all viral promoters in a cascade fashion. That in1814 fails to produce detectable viral proteins following HS of latently infected ganglia affords a unique opportunity to directly examine VP16 promoter function in the absence of other viral proteins. Thus activation of the VP16 promoter following a reactivation stimulus in neurons latently infected with in1814 would provide strong support that transcription of the VP16 gene can be upregulated in the absence of other viral proteins during the earliest stages of exit from latency. To test this hypothesis mutant in1814VP16pLZ was generated as detailed in methods. The in vivo phenotypes of this mutant (replication, establishment of latency and reactivation) were not different than in1814 (not shown). Mice were infected with in1814VP16pLZ and maintained for 20 days pi. Groups of mice were subjected to HS and at 22 hr post HS ganglia were removed and processed to detect b-gal activity. No neurons were positive in 20 TG from infected untreated animals. However, 5/20 of the ganglia from treated mice contained one or more neurons positive for b-gal post HS, demonstrating that the VP16 promoter had been activated in these neurons. In additional studies it was observed that activation of this promoter post HS becomes increasingly rare as time progresses which parallels the ability to detect VP16 protein (not shown).

## Discussion

Successful completion of the complex in vivo life cycle of both herpes simplex virus type 1 and type 2 requires activation of the viral lytic cycle from the latent viral genome. Since HSV latency is characterized by the absence of all detectable lytic viral proteins, the lytic cycle must start de novo, i.e. without the viral proteins normally carried into a cell. These proteins, among other functions, facilitate activation of transcription from the viral genome. Studies performed nearly two decades ago led to the formative conclusion that VP16 does *not* coordinate the exit from latency [15],[16],[72]. Using new approaches we have revisited this important issue and now demonstrate that the transactivating function of VP16 is indeed requisite for HSV reactivation in vivo. The functional requirement for VP16 is very early in the transition from the latent to the lytic cycle, as in its absence the latent viral genome cannot advance to the production of lytic viral proteins. In contrast, elimination of “immediate early” and “early” viral functions previously considered critical for the initiation of reactivation has no measurable effect on lytic viral protein expression at this early stage in reactivation 25,28. We show that replacing the VP16 promoter with another leaky late gene promoter exposes unique regulatory properties of the VP16 promoter in the context of infection in neurons in vivo. The VP16 promoter is responsive to reactivation stimuli in the absence of other viral functions and robust expression of VP16 from the latent viral genome is observed in neurons in the absence of viral functions normally required for efficient late gene expression. These data argue that the expression of VP16 during reactivation diverges from the established late expression pattern of this gene. Our findings lead to the hypothesis that in the context of the latently infected neuron, the expression of VP16 is a critical initiating event, coordinating the activation of the viral IE genes which results in productive entry into the viral lytic cycle.

The differing outcomes and thus conclusions regarding the role of VP16 in reactivation stem primarily from the models of reactivation utilized. In previous studies utilizing in1814 in the mouse ocular or footpad models reactivation was evaluated ex vivo by removing ganglia from the latently infected animal and culturing the tissue (explanting) for >21 days [16],[17]. Recent studies demonstrate that axotomy and explant result in rapid and progressive changes in the gene expression and physiological states of neurons and other cells not observed in ganglia following an in vivo stress resulting in reactivation [24]. These changes can obviate the need for and obscure the roles of viral genes important for the reactivation process [25]. O'Hare suggested that VP16 might indeed play a pivotal role during the early stages of reactivation, proposing that explantation of ganglia into culture might overcome the requirement for VP16 [26], thus explaining reports that in1814 reactivated ex vivo [15]–[17]. Our results confirm this hypothesis.

Quantitative analyses of both latency and reactivation at the single neuron level have increased understanding of the relationships between viral replication, the number of latently infected neurons, and the probability of reactivation [4],[25],[27],[28],[56],[69],[77],[79].Viral replication within the trigeminal ganglia (TG) is required for maximizing the number of latent infections [66] and mutants that replicate poorly in the TG generally establish latency very inefficiently [25],[28]. In previous studies mutant in1814 seemed to be an exception as no replication of in1814 was detected in sensory ganglia [16],[72] and yet latency was established very efficiently as determined by the detection of the latency associated transcripts [16],[72]. However, with the addition of HMBA to the plaque assay, it is clear that in1814 does replicate in the TG. The mutant 17VP16Δ422, however, is severely restricted in TG and establishes very low levels of latency as would be predicted by its limited replication. It seems likely that mutated VP16 protein produced by in1814 still retains some ability to transactivate IE genes, perhaps through elements other than the TAATGARAT. VP16 has been shown to transactivate through the GCGGAA element in IE gene promoters and this activity is independent of transactivation through the TAATGARAT element [80],[81]. Despite the low levels of latency established by 17VP16Δ422 this mutant did reactivate in 67% of the ganglia placed into explant culture within 15 days, a finding that further emphasizes the differences inherent in the in vivo and ex vivo reactivation models.

In vivo reactivation is a tightly regulated event in that following induction the lytic cycle is engaged in ∼0.05% of neurons harboring the latent genome [4]. Viral infection is confined to the individual neurons undergoing reactivation (usually 1–5 neurons per TG) with no spread of infection within the ganglion and consistent with this, it is characterized by low levels of infectious virus in the ganglion [3],[5],[82]. Reactivation of HSV in vivo has been characterized most extensively following hyperthermic stress, however other induction triggers, including ultraviolet light irradiation [3],[6] show a similar outcome. In contrast, in explanted ganglia virus infection spreads unchecked, accounting for the extremely high titers of virus recovered [24]. The limited production of virus in vivo raises the issue of sensitivity and the possibility that the differing reactivation outcomes observed merely reflect differences in detection of the infectious virus produced in the ganglia, a consequence of reduced plaquing efficiency of in1814 compared to 1814R or 17syn+ [40]. This question could not be resolved without a different approach for detecting the exit from latency, one that did not depend on the detection of infectious virus. The need for such an assay has long been recognized [31],[83],[84] and an in situ immunohistochemical method in whole ganglia that permits the detection of lytic phase viral proteins in the very rare neurons that exit latency was employed for this purpose [4],[24],[25],[28],[55],[78]. This assay makes it possible to evaluate the ability of viruses that are blocked or impaired at later steps in the replicative cycle, to exit latency. This assay revealed that the failure to detect infectious virus in the ganglia latently infected with in1814 following induction was not merely an issue of sensitivity but an actual failure of latent in1814 genomes to exit latency. The implications of this observation are profound in that our attention is directed toward the regulation of VP16 from the latent genome as a critical interface between neuronal responses to stress and entry into the lytic cycle.

An important question relating to the mechanism of reactivation is whether this phenotype is unique to VP16 transactivating function or whether other viral functions are required at this earliest stage in the reactivation process. Two hypotheses framed thinking about how the lytic cycle was initiated from the latent genome without VP16 function. The first suggested that ICP0 served this function [18]–[21] and the second proposed that limited viral DNA replication is required for and precedes efficient expression of IE genes [22],[23]. Using the same approaches utilized for the analysis of in1814, the role of these viral functions in reactivation were examined. In the absence of ICP0 function, initiation of reactivation as evidenced by lytic viral protein expression was not measurably different from that in the rescue or parental strain. Thus in contrast to VP16 transactivating function, ICP0 is not essential for these very early events. A similar result was observed when viral DNA replication were blocked either pharmacologically or genetically. In both cases, viral proteins but not infectious virus was detected, confirming the roles of these viral functions for the progression to infectious virus production [25],[28]. These studies emphasize that VP16 appears to play a unique role in the earliest stages of reactivation to coordinate IE gene expression and thereby entry into the lytic cycle. Further evidence that VP16 functions early in the process of reactivation to upregulate IE genes through the TAATGARAT motif comes from our finding that adding back the TAATGARAT sequences to the ICP0 promoter in mutant ΔTfi restored the ability of this mutant to reactivate in vivo. This is in keeping with a requirement for ICP0 for progression to infectious virus production and the TAATGARAT element for proper expression of ICP0 during reactivation in vivo.

The extreme rarity of viral reactivation at the neuronal level might be explained if stress induced changes occur only in very rare neurons, or if only a few latently infected neurons contain viral genomes capable of reactivation, but neither is the case. Stress does induce global changes in the chromatin structure of latent viral genomes [85]–[90]. To be detected such changes must occur on the majority of latent viral genomes, while only a very few viral genomes exit the latent state. Thus, the alterations in chromatin measured may or may not be necessary but are *not* sufficient to precipitate viral reactivation. Many latently infected neurons are capable of reactivation as evidenced by semi-quantitative assays performed on dissociated latently infected ganglia [91]. Likewise, over a period of a few days, 100s of neurons produce viral proteins (exit the latent state) in TG axotomized and explanted into culture in the presence of the antiviral drug acyclovir (which prevents virus replication and spread within the TG) [24],[28]. Further, we have determined that viral reactivation can be induced repeatedly (30 times over 10 months) in the same mice in vivo without reduction in the frequency of reactivation (unpublished).

Reactivation from latency has been traditionally thought to be the result of changes in host neuronal transcriptional regulators induced by systemic stress which then directly stimulate the activation of one or more viral promoters. However, it is difficult to reconcile the extreme rarity of these reactivation events with this simple genetic model. For this model to be correct the stress induced signals that initiate reactivation from latency must only occur in very rare neurons at any given time, and since neurons do not survive viral reactivation [3],[5],[78], these same rare changes would have to occur in a new rare subset of neurons as each virus reactivation event occurs through time. The extremely low frequency of reactivation suggests a model of stochastic derepression of VP16 in the presence of positive transcription factors [92]. This type of model would predict that the transcription and/or translation of viral genes is extremely repressed and that viral reactivation is essentially an extremely rare and seemingly random event precipitated when the amount of VP16 protein present in a given neuron reaches a level adequate to initiate the cascade of lytic viral gene expression.

The hypothesis that VP16 functions in conjunction with host cell proteins as a regulatory switch, promoting the lytic cycle when VP16 is present and latency in its absence has been proposed by many investigators [13],[15],[26],[32],[83],[93]. Sears and Roizman first proposed that the VP16 protein in the tegument may be inefficiently transported the distance through the axon to the cell body, thereby promoting latency [15]. Although their early attempts to support this hypothesis experimentally were not successful, there is support for the notion that the VP16 equivalent in pseudorabies virus is dissociated from the nucleocapsid prior to transport to the neuronal cell body [43]. Importantly, our results imply that VP16 generated and packaged into virions during surface replication is not sufficient to efficiently activate IE genes in TG neurons.

**Figure 7:**
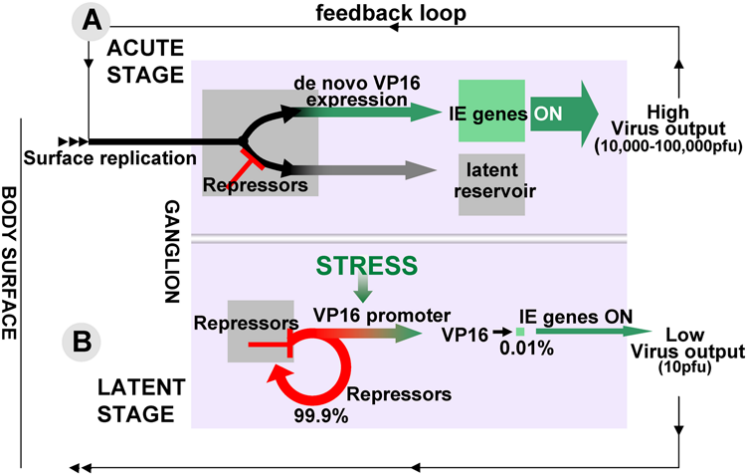
The regulation of expression of VP16 controls the balance between lytic and latent infection of sensory neurons at all stages of the virus infection cycle. (A) Shown is a schematic of the role of de novo VP16 expression during acute infection of the trigeminal ganglion. As detailed in the text, we hypothesize that VP16 protein is not transported efficiently to neuronal cell bodies and that sequences present in the VP16 promoter direct the de novo expression of VP16, which overcomes repressors (e.g. riboregulators) to initiate lytic infection during the acute stage of viral replication. (B) We hypothesize that following stress the de novo synthesis of VP16 initiates a feedback loop with the IE genes that results in viral reactivation in one or a very few of the 6,000 latently infected neurons present in the ganglion.

We propose that neuron driven regulation of VP16 orchestrates its de novo synthesis which is central to coordinated entry into the lytic cycle in neurons and the balance between the lytic and latent viral programs (Figure 7). During acute infection, virus replication at the body surface feeds virions into the axons of innervating sensory neurons (Figure 7A). Data support the notion that VP16 is not transported efficiently to the neuron nucleus and in its absence the latent transcriptional pathway is engaged. De novo expression of VP16 is then required for the virus to enter the lytic pathway in these neurons and VP16 is only expressed when repressors are overcome. Repressors that operate during the acute stage of infection are likely to include riboregulators encoded by the latency associated transcript locus (LAT) [94]–[96]. Expression of the LAT locus is required for ∼65% of the latent infections established [27],[79], and in the absence of LAT transcription, half of the neurons destined to be latently infected enter the lytic pathway and die [27]. If sufficient VP16 is expressed to coordinate IE gene expression, a positive feedback loop overcomes repression, the neurons becomes lytically infected and the virus produced spreads both within the ganglion as well as back down to the surface. Efficient virus replication at the surface and within TG is required to maximize the number of latent infections established [66]. Acute virus replication ends by about 10 days pi and during the period between 10 and 40 days pi latency becomes consolidated (Figure 7B), perhaps through chromatinization of the viral genome [85], [88]–[90],[97],[98], the recently proposed immune mediated non-lethal inhibition of virus production once the exit from latency has been initiated in neurons [99], and/or the build up of riboregulators [94]–[96],[100],[101], and fewer latently infected neurons respond to stresses that induce viral reactivation [4]. By 40 day pi about 0.05% of latently infected neurons show evidence of viral reactivation following HS whereas latency is maintained in the other 99.95% [4],[56],[69]. We hypothesize that stress induces the de novo production of VP16, which only rarely reaches levels sufficient to coordinate activation of the IE genes that overcome repressive factors and initiate the lytic transcription program.

## Materials and Methods

### Viral strains/mutants and stock production

Stocks of HSV-1 strain 17syn+ and the mutants employed in this study were generated in rabbit skin cell (RSC) monolayers and the viral titers were determined by serial-dilution plaque assay [5],[102]. The wild type HSV-1 strain 17syn+ was originally obtained from John H. Subak-Sharpe at the MRC Virology Unit in Glasgow, Scotland. The generation and characterization of the VP16 transactivation deficient mutant in1814 and its genomically restored counterpart, 1814R, have been described [16],[40]. Where indicated, the plaquing efficiency of in1814 was enhanced by the addition of 3 mM hexamethylene bisacetamide (HMBA, Sigma) as described [74],[103]. The mutant ΔTfi and its genomically restored counterpart, ΔTfiR were a kind gift of David Leib, Washington University, and have been described in detail [29],[104].

### Construction of new viral mutants

All restriction enzyme sites and base pair numbering are referred to as the corresponding positions in the published HSV-1 sequence of strain 17syn+ [105],[106] as present in Genbank (NID g1944536).

### Mutant VP5p/VP16

A 649 bp DNA molecule was synthesized (Blue Heron) in which the VP16 promoter and 5′UTR (bp 105,435 to 105,107) was converted to the promoter and 5′UTR of VP5 (bp 40,659 to 40,812), flanked by appropriate sequences and recombined into strain 17Syn+ The promoter structures of 5 independently derived mutants were confirmed by Southern blot analysis and DNA sequencing (not shown). Wild type UL49-UL48 sequences were recombined with VP5p/VP16-1 to generate the genomically restored variant VP5p/VP16-1R as described [27],[79].

### Mutant 17VP16Δ422

Mutant 17VP16Δ422 was generated in strain 17syn+ to be similar to mutant V422, in which the carboxy-terminal acidic transactivation domain of VP16 was deleted in strain KOS [107]. The eGFP gene driven by the b-actin promoter was inserted at the SacI site at bp 103,808 in the VP16 ORF in the orientation opposite to VP16, truncating the protein at amino acid 422 and facilitating selection of the mutants. 3 mM HMBA was added to the culture medium to increase the plaquing efficiency of the VP16 mutants [103]. Following low MOI infection of RSC, mutants displayed a reduced capacity to replicate and plaque which was increased 40 fold in the presence of HMBA. Wild type UL49-UL48 sequences were recombined with 17VP16Δ422 to generate the genomically restored variant 17VP16Δ422R as described [27],[79].

### Mutant ΔTfi+TAATGARAT

Truncation of the ICP0 promoter at −145 just prior to the TAATGARAT element resulted in an ICP0 promoter that drives expression efficiently in cells and tissues in vivo with the exception of TG neurons, where this promoter fails to function [55]. Using this information, a construct was generated that restored sequences to −172 in the ΔTfi ICP0 promoter including 27 additional bases (CGTGCATGCTAATGATATTCTTTGGGGG) that contain the functional TAATGARAT of ICPO (underlined). This construct was recombined into both ICP0 promoters in the mutant ΔTfi to generate the ICP0 promoter mutants termed ΔTfi+TAATGARAT. Independently derived mutants were generated and characterized and all replicated as well as wild type in RSC and in mice in vivo (not shown).

### Mutants 17VP16pLZ and in1814VP16pLZ

Mutants 17VP16pLZ and in1814VP16pLZ express the E. Coli beta-galactosidase gene (b-gal) from a 423 bp promoter/5′UTR fragment of the VP16 gene (105,108-105,534 bp) inserted into the intergenic region between glycoprotein J (gJ) and gD in strain 17Syn+, or the VP16 transactivation mutant in1814 respectively. A single base mutation (G>A) was introduced at 138,045 on the viral genome to generate an EcoRV site in the intergenic region between gJ and gD. The promoter/reporter cassette (terminated by bi-directional SV40 polyadenylation signals) was cloned in the orientation opposite that of the viral gJ and recombined in to the genome [55]. Six independently derived mutants replicated as well as wild type in RSC and in mice in vivo (not shown). Wild type levels of gJ and gD mRNA of the expected sizes were produced by the mutants (not shown).

### Inoculation of mice

All procedures involving animals were approved by the Children's Hospital or University of Cincinnati Institutional Animal Care and Use Committee and were in compliance with the *Guide for the Care and Use of Laboratory Animals*. Animals were housed in American Association for Laboratory Animal Care-approved quarters. Male, outbred, Swiss Webster mice (22–25 grams in weight, Harlan Laboratories) were used throughout these studies. Prior to inoculation, mice were anesthetized by intraperitoneal injection of sodium pentobarbital (50 mg/kg of body weight). A 10 ul drop containing the amount of virus as detailed in the text was placed onto each scarified corneal surface. In some experiments the inoculum titer was altered to achieve uniform levels of latent infections as previously detailed [56],[66]. In preliminary experiments we determined that inoculation of mice with 2×10^5^ pfu of strain 17syn+ or in1814R and 8×10^5^ pfu of in1814 resulted in similar levels of latency as shown in Figure 4B. In these preliminary studies mice infected with in1814 at 2×10^5^ pfu were examined. In these mice as in those receiving the higher imput titer no exit from latency was detected.

### Replication in vivo

Mice infected as above were euthanized at the indicated times post infection and tissues from three mice from each inoculation group were individually assayed for virus as previously detailed [49].

### Quantification of latent infections by contextual analysis of latency

Additional mice from the groups infected as above were maintained for at least 40 days pi. Enriched neuron populations were obtained and individual neurons were assayed for the presence of the latent viral genome as described [77],[79].

### Quantification of viral genomes by real time PCR assay

Isolation and quantification of total DNA from TG and quantification of total viral genomes by real time PCR was performed as detailed previously [28].

### In vivo reactivation

Latent HSV was induced to reactivate in the ganglia of mice in vivo using hyperthermic stress (HS) and at 22 hours post induction TG were assayed for infectious virus as detailed previously [5].

### In vitro explant reactivation

Latently infected ganglia were aseptically removed and placed into Minimum Essential Media (MEM) supplemented with 5% newborn calf serum and incubated at 37°C in a 5% CO_2_ incubator. At the indicated times post explant, ganglia were homogenized and assayed for infectious virus as for reactivation in vivo [24].

### Antibodies and immunohistochemistry

Colocalization of b-gal activity and HSV lytic viral proteins was carried out by first histochemically staining whole TG by incubation in x-gal (Sigma) followed by paraffin embedding of TG, sectioning and immunohistochemical detection of viral proteins. Where indicated, HSV proteins were also detected in whole ganglia using whole ganglia immunohistochemistry (WGIHC). Primary antibodies utilized include rabbit anti-HSV (AXL237, Accurate), rabbit anti-VP16 antibody (clonetech), and secondary antibody utilized was HRP labeled goat anti-rabbit (Vector). These methods and the dilutions and characterizations of antibodies utilized have been detailed extensively in previous reports [4],[55].
